# Cycle training and factors associated with cycling among adolescents in England

**DOI:** 10.1016/j.jth.2019.100815

**Published:** 2020-03

**Authors:** Ailsa McKay, Anna Goodman, Esther van Sluijs, Christopher Millett, Anthony A. Laverty

**Affiliations:** a Public Health Policy Evaluation Unit, School of Public Health, Imperial College London, UK; b Faculty of Epidemiology and Population Health, London School of Hygiene and Tropical Medicine, UK; c Centre for Diet and Activity Research (CEDAR), MRC Epidemiology Unit, University of Cambridge School of Clinical Medicine, UK

**Keywords:** Cycling, Adolescents, Cycle training

## Abstract

**Background:**

Cycling has the potential to encourage physical activity as well as advancing societal goals such as reducing carbon emissions; encouraging cycling is therefore a policy goal in many contexts. We analysed individual level data from the whole of England on factors associated with cycling among adolescents, including cycle training delivered by the age of 11 years in primary schools.

**Methods:**

Data came from the nationally representative Millennium Cohort Study collected when participants were aged 13–15 years (adolescents). We assessed frequency of cycling at least once per week (regular cycling) and used logistic regression to assess how this differed across characteristics including demographic, health and environmental factors, as well as receiving cycle training (‘*Bikeability’*) in primary school.

**Results:**

We found that 21.0% of adolescents cycled at least once per week. In fully adjusted analyses, this was more common among boys than girls (32.5% vs. 9.4%, p < 0.001), and those in rural areas than urban areas (24.9% vs. 20.3%, p < 0.001). Adolescents in areas with higher prevalence of adult cycle commuting were more likely to cycle regularly (26.1% in high cycling areas vs. 19.3% in low cycling areas, p < 0.001). Participants offered cycle training in primary school were not more likely to cycle regularly as adolescents (21.7% vs. 22.3%, p = 0.528).

**Discussion:**

Approximately one in five adolescents in England cycles regularly, although being offered cycle training in primary school was not linked to greater cycling. Many of the factors associated with adolescent cycling are similar to those for adults and adolescents are more likely to cycle in areas with higher levels of adult cycling.

## Highlights

• Approximately one in five cycles regularly.
• This was more common among boys, in rural areas and in areas with higher levels of adult cycle commuting.
• Receiving cycle training at age 10/11 years is not linked to more cycling as an adolescent.

## 1 Introduction

Physical activity-promoting policies and interventions have long been recommended to improve a wide range of physical health outcomes and wellbeing (World Health Organization, 2010; US Department of Health and Human Services, 1997). Increased use of cycling has been cited as having the potential to encourage more physical activity and advance societal health and environmental goals (Roth et al., 2012; Sahlqvist et al., 2012). Nonetheless, as with other transport options, there are a number of influences on cycling at the area, school, family and individual level (Panter et al., 2010). Understanding the factors associated with cycling is therefore important in promoting both uptake and equality of access.

Among previously reported predictors of cycling in children and young people are convenience of alternative forms of transport, environmental factors including bicycle path provision and safety concerns (Ducheyne et al., 2012; Carver et al., 2005; Panter et al., 2013). In common with cycling among adults, shorter distances are linked to being more likely to cycle among children and young people, as is use of motorized modes being inconvenient [8]. Gender and socioeconomic status are also important, as are peer support, and familiarity with others in the neighbourhood (Panter et al., 2010, 2013; Ducheyne et al., 2013). Parental perceptions of cycling skills and familiarity with different traffic situations have been found to be linked to more cycling (Ghekiere et al., 2014). Enhancing such confidence and skills in cycling is the aim of cycle training, which despite being reasonably widespread, particularly in countries where cycling is more common, has limited evidence of impact (Pucher and Buehler, 2008; Sersli et al., 2018). Some studies have demonstrated impact on knowledge, but there is less evidence for effect on attitudes (such as considering cycling to be a safe activity) and behavior (such as greater cycling frequency) (Pucher and Buehler, 2008; Sersli et al., 2018).

In England, the *Bikeability* cycle training programme was introduced by the Department of Transport (DfT) in 2007 (Department of Transport, 2012). The programme is offered to children in participating schools at ages 9–11 years, and aims to facilitate safer and more frequent cycling by enhancing relevant knowledge, skills and confidence (Department of Transport, 2012; England, 2010). Evaluation of its impact on cycling behaviour at age 10–11 years (i.e. around the time of training delivery) have produced mixed findings, with one study indicated little effect on cycling frequency or independent cycling but a more recent smaller evaluation suggesting increased cycling (Goodman et al., 2016; SQW, 2019). However, impact at later follow-up (when potentially limiting factors such as availability of parental supervision and limited confidence may be less applicable) has not been reported. We therefore aimed to assess whether cycle training was linked to more cycling among adolescents and the socio-demographic and environmental factors associated with cycling regularly among adolescents.

## 2 Methods

We used data from the Millennium Cohort Study (MCS), an ongoing longitudinal study which collects data from UK children born between September 2000 and January 2002 (Fitzsimons, 2017). The original sample was based on a stratified cluster framework, with oversampling of smaller population groups including those who lived in economically deprived areas and those of ethnic minority backgrounds (Fitzsimons, 2017). Data collections involved interviews with caregivers and participants and our sample was based on the 11,726 13-15 year-olds who participated in 2014/15. We restricted our analyses to the 7634 participants resident in England, as *Bikeability* data and some geographic variables were not available for the other UK nations.

The MCS asked participants about cycling frequency was assessed by asking participants the question, ‘*How often do you use a bicycle? Please include travel to and from school*’. There were eight possible responses: ‘*every day or almost every day*’; ‘*several times a week*’; ‘*once or twice a week*’ ‘*at least once a month*’; ‘*every few months*’; ‘*at least once a year*’; ‘*less often or never*’; ‘*do not use a bicycle.*’ From these, we generated a outcome variable of at least per week, referred to as 'regular cycling’.

More detailed information about data collection and derivation of exposures is available in Supplementary File 1. We included information on self-reported age; gender; self-defined ethnic background; care-giver reported household income category; and geographical region of residence (English Government Office Region). A categorical BMI variable was constructed from objective measures of weight and height. Self-rated general health (described as ‘excellent’, ‘very good’, ‘good’, ‘fair’ or ‘poor’) and weekly physical activity (≥1-hour of moderate-to-vigorous activity on 0–2, 3–4 or ≥5 days/week) variables were generated. Additional variables described the urban/rural status of the participant's home address (Bibby and Shepherd, 2004), and proportion of adult cycle commuting in local area (categorised as <2, 2-3.9, 4-5.9 and 6+%) from the 2011 UK Census (Office for National Statistics, 2011). These latter two variables were defined using Lower Super Output Areas, which are geographical areas containing around 1500 individuals on average, and used by the Office for National Statistics for linking people to their local area data. We used data from the Department of Transport to create a variable describing whether a participant attended a primary school where *Bikeability* training was offered (using data from when participants were 10/11 years old), and refer to this here as being offered cycle training (15). For some participants whether their primary school offered *Bikeability* training was unknown, primarily because *Bikeability* data was unavailable for schools in London, as well as some participants being home-schooled.

Missing data was low, at 4.3% for BMI values and otherwise ranging from 0 to 0.7% for all other predictor variables. Overall, 94.9% of participants had no missing data on any variable. For the modest amount of missing data, we performed multiple imputation of missing data under a missing at random assumption, using five imputations in chained equations. We included in our imputation model the outcome and all explanatory variables in the model, plus weight status at age 10/11 in order to facilitate imputation of current weight status. We performed this imputation in order to increase study power, although we note that our substantive findings were very similar in a complete case analysis that excluded children with missing data.

We used logistic regression to explore associations of our potential factors associated with regular cycling, and we present odds ratios both unadjusted and adjusted for all included exposures in one model (fully-adjusted). We also employed tests for differences by trend for ordered categorical variables developed by Cuzick which assess if there is a trend across categories of individual variables. We performed a sensitivity analysis of whether participants reported completing any cycle training (any training ‘delivered by a recognised trainer’ that included road-based tuition), rather than being *offered Bikeability* training in their school. The disadvantage of this second measure (and the reason we only used it in a sensitivity analysis) is it is potentially more open to selection bias. For example, children who are already interested in cycling are more likely to seek out training, alternatively, children who are not confident at cycling may seek out such training. So we are using two slightly different measures of cycle training: being offered cycle training, which is based on school level data, and reporting completing cycle training, which is self-reported at the individual level.

In all analyses, we applied survey weights which were provided by the MCS team were applied to protect the representativeness of the sample, by correcting for the sampling strategy, attrition and non-response (Fitzsimons, 2017).

As this study was an analysis of pre-existing secondary data with no identifiable information, ethical approval was not required. Participants gave informed consent for their data to be used for research purposes. The MCS study as a whole has ethical approval from the Yorkshire and Humber ethics committee (further details here https://cls.ucl.ac.uk/).

## 3 Results

Among the 7634 individuals identified as eligible for inclusion in our study 49.7% were female, and age at interview ranged from 13 to 15 years (75.0% 14 years). (Table 1). We found that 3595 (47.1%) had been offered *Bikeability* training, and 3766 (50.8%) completed cycle training.

**Table 1:** Characteristics of sample of adolescents in Millennium Cohort Study (data collected 2014/15).

		n (total n = 7634)	% *
Age (years)	13	1896	23.9
	14/15	5738	72.3
Gender	Male	3857	48.6
	Female	3777	47.6
Ethnic background	White	5802	73.1
	Mixed	96	1.2
	South Asian	1148	14.5
	Black	382	4.8
	Other	201	2.5
Body mass index category	Underweight or healthy weight	4425	55.8
	Overweight	1678	21.1
	Obese	1044	13.2
Self-rated general health	Excellent	923	11.6
	Very good	2712	34.2
	Good	2743	34.6
	Fair	869	11.0
	Poor	149	1.9
Weekly physical activity	0–2 days	2166	27.3
	3–4 days	2513	31.7
	5 or more days	2736	34.5
Household income category	Highest quintile	1813	22.9
	Quintile 2	1665	21.0
	Quintile 3	1433	18.1
	Quintile 4	1286	16.2
	Lowest quintile	1437	18.1
Region	North East	326	4.1
	North West	942	11.9
	Yorkshire	905	11.4
	East Midlands	639	8.1
	West Midlands	913	11.5
	East of England	860	10.8
	South East + London	2374	29.9
	South West	675	8.5
Urban/rural area	Urban	6407	80.8
	Rural	1219	15.4
Proportion adult cycle commuting in local area (in 2011)	<2%	4154	52.4
	2 - 3.9%	2173	27.4
	4-5.9%	685	8.6
	6%+	614	7.7
Offered Bikeability at primary school	No	2708	34.1
	Yes	3594	45.3
	Unknown	1332	16.8
Self-reported completion of cycle training	Yes	3644	45.9
	No	3767	47.5

*NB. Reported percentages disregard missing data, which is described in the main text (and imputed for regression analyses).

We found that 21.0% of all participants cycled at least once per week (Table 2). In adjusted analyses cycling was less common among girls (9.4%) than boys (32.5%) (Adjusted Odds Ratio (AOR) = 0.24, 95% Confidence Interval 0.21–0.28) and more common among White participants than those of other ethnic backgrounds. Cycling was more common among those in rural areas compared with urban areas (AOR = 1.45, 1.17–1.80), and there was statistically significant evidence of a dose-response relationship between adolescent cycling and prevalence of cycle commuting among adults (p for trend < 0.001). There was statistically significant evidence of a dose-response relationship with more physically active participants being more likely to cycle regularly (p for trend <0.001) and those with poorer self-rated health being less likely to cycle regularly (p for trend <0.001). There was statistically significant evidence of a dose-response relationship with participants in lower income households were more likely to cycle regularly (p for trend 0.002). We did not identify statistically significant differences between regions, and regular cycling varied from 17.8% in London and the South East to 26.1 in the East Midlands. Being offered *Bikeability* in primary school was not associated with cycling regularly (AOR = 0.94, 0.80–1.10), although sensitivity analyses of self-reported participation in cycle training was (AOR = 1.46, 1.26–1.69).

**Table 2:** Factors associated with weekly cycling.

		Weekly cycling (% in category)	UOR (CI)	p	AOR (CI)	p
Age (years)	13	20.2	ref	0.261	ref	0.225
	14/15	21.3	1.10 (0.93–1.29)		1.11 (0.94–1.31)	
Gender	Male	32.5	ref	<0.001	ref	<0.001
	Female	9.4	0.21 (0.19–0.25)		0.24 (0.21–0.28)	
Ethnic background	White	22.4	ref	<0.001	ref	<0.001
	Mixed	17.4	0.67 (0.37–1.12)		0.64 (0.34–1.21)	
	South Asian	17.6	0.69 (0.56–0.86)		0.67 (0.5–0.9)	
	Black	13.4	0.47 (0.31–0.72)		0.44 (0.28–0.71)	
	Other	16.8	0.69 (0.47–1.01)		0.75 (0.48–1.17)	
BMI category	Underweight/healthy weight	21.4	ref	0.066	ref	0.092
	Overweight	21.5	1.03 (0.88–1.20)		1.21 (1.02–1.44)	
	Obese	20.1	0.80 (0.64–0.98)		1.06 (0.85–1.33)	
Self-rated general health	Excellent	33.4	ref	<0.001	ref	<0.001
	Very good	22.7	0.62 (0.50–0.77)		0.79 (0.64–0.99)	
	Good	17.8	0.44 (0.36–0.54)		0.66 (0.53–0.82)	
	Fair	13.8	0.31 (0.23–0.43)		0.5 (0.36–0.7)	
	Poor	15.4	0.36 (0.19–0.65)		0.67 (0.35–1.3)	
Weekly physical activity	0–2 days	10.1	ref	<0.001	ref	<0.001
	3–4 days	17.3	1.96 (1.60–2.40)		1.8 (1.44–2.25)	
	5 or more days	33	4.38 (3.61–5.32)		3.33 (2.69–4.12)	
Household income category	Highest quintile	20.3	ref	0.503	ref	0.019
	Quintile 2	20.2	1.01 (0.86–1.19)		1.02 (0.85–1.22)	
	Quintile 3	20	1.08 (0.88–0.32)		1.19 (0.96–1.48)	
	Quintile 4	23.1	1.19 (0.97–1.48)		1.47 (1.16–1.88)	
	Lowest quintile	22	1.08 (0.88–0.33)		1.46 (1.15–1.85)	
Region	North East	21.2	ref	0.005	ref	0.014
	North West	21.2	0.91 (0.63–1.34)		0.95 (0.63–1.42)	
	Yorkshire	21.8	0.95 (0.53–1.68)		1.03 (0.59–1.8)	
	East Midlands	26.1	1.35 (0.91–2.01)		1.35 (0.88–2.06)	
	West Midlands	21.9	1.11 (0.73–1.70)		1.29 (0.83–1.99)	
	East of England	23.6	1.12 (0.76–1.68)		1.19 (0.76–1.86)	
	South East + London	17.8	0.77 (0.53–1.13)		0.92 (0.6–1.43)	
	South West	21.6	0.94 (0.63–1.39)		0.8 (0.53–1.22)	
Urban/rural area	Urban	20.3	ref	<0.001	ref	0.001
	Rural	24.9	1.37 (0.15–1.63)		1.42 (1.14–1.75)	
Proportion adult commuting in local area (in 2011)	<2%	19.3	ref	0.131	ref	0.003
	2 - 3.9%	22.1	1.17 (0.98–1.40)		1.29 (1.06–1.58)	
	4-5.9%	23.3	1.16 (0.92–1.46)		1.43 (1.1–1.84)	
	6%+	26.1	1.39 (0.98–1.96)		1.75 (1.25–2.45)	
Offered Bikeability at primary school	No	22.4	ref	0.036	ref	0.508
	Yes	21.6	0.96 (0.82–1.12)		0.94 (0.80–1.10)	
	Unknown	16.6	0.71 (0.55–0.92)		0.84 (0.60–1.17)	

UOR: unadjusted odds ratio; CI: confidence interval; AOR: adjusted odds ratio (i.e. from multivariable analysis using model incorporating all other variables in table), BMI: body mass index. Total n = 7634.

Unadjusted analyses produced similar results, although they did suggest a stronger association between weekly physical activity and cycling (e.g. AOR of 4.38 for those partaking in physical activity five or more times per week, compared with an AOR of 3.33 in adjusted analyses).

## 4 Discussion

This representative study of adolescents in England found that approximately one in five cycles regularly but that this was more common among boys, White children, those partaking in other physical activity, as well as those in rural areas and in areas with higher levels of adult cycle commuting. We also found that being offered cycle training at primary school was not associated with cycling among adolescents, although self-reported completion of cycle training was.

The associations we observed for gender, ethnicity and socioeconomic status are in keeping with previous findings (all from high-income country contexts) and are similar to those identified for adult cycling (Panter et al., 2010, 2013; Biehl et al., 2018; Carver et al., 2015; Goodman and Aldred, 2018). Nonetheless there is less understanding of interventions that could specifically encourage cycling among low-uptake groups. Indeed previous work suggests that some current cycle policies may have served to enhance rather than reduce socio-demographic inequalities in access to cycling (Ogilvie and Goodman, 2012; Lee and S.I.Jones, 2017). The association observed here between rural living and cycling may appear out of keeping with previous suggestion that greater travel durations (likely to occur more frequently in rural settings) are negatively associated with cycling frequency (Panter et al., 2013; Larouche, 2015), but is potentially explained by the inclusion of cycling for non-travel reasons within our outcome definition. It could also reflect the impact of factors such accessibility of alternative transport options, which we were unable to account for here. The observed association between adolescent cycling and extent of adult commuting by cycling is similarly potentially explainable by unmeasured factors previously found to be associated with cycling behaviour - including cycling infrastructure and other elements of the road environment, trip distances and hilliness, weather conditions, and attitudes towards cycling (Panter et al., 2010; Ducheyne et al., 2012; Hume et al., 2009; Scriven, 2012). Such factors may also be relevant to the observed association between physical activity levels and cycling, but as our physical activity measure incorporated cycling, this association should be interpreted cautiously.

That offer of *Bikeability* training did not show a clear association with increased cycling frequency is consistent with previous studies of both *Bikeability* and cycle training more generally (Goodman et al., 2016; Richmond et al., 2014) Adolescents who reported participating in cycle training were more likely to cycle but this was not the case for adolescents who attended a primary school which offered such training. There was some overlap between these responses, and 60.7% of children attending a primary school which offered *Bikeability* reported that they had received training. Previous analyses have suggested that schools in more deprived areas were less likely to offer cycle training, and it may be that such programmes are partyl reflecting the more affluent profile of children taking part (Goodman et al., 2016). The findings here reinforce previous suggestions that the link between such training and cycling is not causal, but reflects unmeasured factors previously found to be associated with cycling, (e.g. prior interest in and attitudes towards cycling, home and social arrangements that facilitate cycling, and bicycle ownership), rather than effect of training *per se* (Goodman et al., 2016).

This study contained information from a large well-established cohort of adolescents from across England and we were able to consider a wide range of potential factors associated with cycling, including demographic, health and environmental factors. However, limitations include a lack of information about some factors understood to be relevant, such as trip distances and alternative transport availability. Previous work also notes potential interactions between different factors (such as whether long distances can reduce confidence in ability to cycle required routes) which we have not considered here (Biehl et al., 2018). It may also be the case that there is some reverse causality with more physically active participants being more likely to cycle. The question regarding cycling frequency also did not differentiate travel and non-travel cycling purposes. Finally, for prevalence of cycling in the local area we have used the last available data for this which is from the 2011 Census, and these levels may have changed since then.

## 5 Conclusion

This study used national data form England to assess whether cycle training was linked to greater cycling among adolescents. We did not find that being offered cycle training before age 11 years was linked to greater cycling, which suggests that any impacts of cycle training programmes in terms of cycling frequency do not extend into adolescence. We found that approximately one in five adolescents in England cycles regularly, and that this was more common among some groups in multivariate analyses. For example cycling was more likely among boys, those in rural areas, those partaking in other physical activity and those in areas with higher levels of adult cycle commuting. Many of these factors associated with adolescent cycling are similar to those for adults and adolescents are more likely to cycle in areas with higher levels of adult cycling.

## Funding

This study is funded by the National Institute for Health Research (NIHR) School for Public Health Research (Grant Reference Number PD-SPH-2015) as well as a NIHR Research Professorship award to Professor Millett (NIHR RP_2014-04-032). The views expressed are those of the author(s) and not necessarily those of the NIHR or the Department of Health and Social Care. The funder had no input in the writing of the manuscript or decision to submit for publication. The NIHR School for Public Health Research is a partnership between the Universities of Sheffield; Bristol; Cambridge; Imperial; and University College London; The London School for Hygiene and Tropical Medicine (LSHTM); LiLaC – a collaboration between the Universities of Liverpool and Lancaster; and Fuse - The Centre for Translational Research in Public Health a collaboration between Newcastle, Durham, Northumbria, Sunderland and Teesside Universities.

## CRediT authorship contribution statement

**Ailsa McKay:** Writing - original draft.

## Declaration of competing interest

AG is a co-investigator on two research projects commissioned by the Department for Transport, and receives funding from the Department for Transport for this work. One project involves evaluating the Cycle City Ambition programme (20164-2021), the other involves building a ‘National Propensity to Cycle’ tool (2015–2019). She has also done paid consultancy work in 2017 and 2019 as an external peer reviewer of an evaluation of the Bikeability scheme commissioned by the Department for Transport. The present research is not funded by the Department for Transport. Other authors have no relevant interests to declare.
