# Supplementary material for: Cycle training and factors associated with cycling among adolescents in England
Source: J Transp Health. 2020 Mar;16:100815. doi: 10.1016/j.jth.2019.100815 (PMC7197752; doi:10.1016/j.jth.2019.100815)
Supplement: Multimedia component 1 [file mmc1.pdf]

Supplementary File 1: Construction of predictor variables

The variables based on the MCS6 questionnaires – and their derivation from the available survey answers – are outlined in the table below.

Variable	Relevant MCS survey	Relevant survey question(s)	Available survey answers	Categorisation
Ethnic background	MCS6 young person questionnaire	What is your ethnic group?	English/Welsh/Scottish/ Northern Irish/British; Irish; Gypsy or Irish Traveller; Any other White background	White
			White and Black Caribbean; White and Black African; White and Asian; Any other mixed/multiple ethnic background	Mixed
			Indian; Pakistani; Bangladeshi	South Asian
			African; Caribbean; Other Black/ African/ Caribbean background	Black
			Chinese; Any other Asian background; Arab; Other ethnic group	Other
Self-rated general health		How would you describe your health generally?	Excellent	Excellent
			Very good	Very good
			Good	Good
			Fair	Fair
			Poor	Poor
Weekly Physical activity		On how many days in the last week did you do a total of **at least an hour** of moderate to vigorous physical activity? By **moderate to vigorous** we mean any physical activity that makes you get warmer, breathe harder and makes your heart beat faster, e.g. riding a bike, running, playing football, swimming, dancing, etc	Every day	≥5 days/week
			5-6 day	
			3-4 days	3-4 days/week
			1-2 days	0-2 days/week
			Not at all	
Ever-receipt of cycle training		Have you ever done any formal cycling proficiency training such as 'Bikeability'? Formal cycling proficiency training is delivered by a recognised trainer and includes tuition on the road.	Yes	Yes
			No	No
Household income category	MCS6 derived variables	Collapses OECD equivalised income variable into quintiles (income derived from series of questions in Household and Parental Questionnaire)	Responses from pan-UK responders categorised into quintiles	Lowest quintile
				Quintile 2
				Quintile 3
				Quintile 4
				Highest quintile
Geographical region of residence (English		Derived from geographical information using postcode lookup.	North East	North East
			North West	North West

Government Office Region)			Yorkshire and the Humber	Yorkshire and the Humber
			East Midlands	East Midlands
			West Midlands	West Midlands
			East of England	East of England
			London	London and South East
			South East	
			South West	South West
Highest academic/ vocational qualification of main caregiver	All MCS sweeps – derived variable	Please tell me which, if any, of the academic qualifications on this card you [^have/have gained since [^date of last main/partner interview]]1?	Higher Degree and Postgraduate qualifications; Post-graduate Diplomas and Certificates; Professional qualifications at degree level e.g. graduate member of professional institute, chartered accountant or surveyor	Postgraduate education
			First Degree (including B.Ed.); Diplomas in higher education and other higher education qualifications; Teaching qualifications for schools or further education (below degree level); Nursing or other medical qualifications (below degree level); NVQ or SVQ level 4 or 5; HND, HNC, Higher Level BTEC/RSA Higher Diploma	First degree or other higher education qualification
			A/AS/S Levels/SCE Higher, Scottish Certificate Sixth Year Studies, Leaving Certificate or equivalent; NVQ or SVQ Level 3/GNVQ Advanced or GSVQ Level 3 OND, ONCM BTEC National, SCOTVEC National Certificate; City & Guilds advanced craft, Part III/RSA Advanced Diploma	Higher secondary qualifications
			O Level or GCSE grade A-C, SCE Standard, Ordinary grades 1-3 or Junior Certificate grade A-C; NVQ or SVQ Level 2/GNVQ Intermediate or GSVQ Level 2; BTEC, SCOTVEC first or general diploma; City & Guilds Craft or Part II/RSA Diploma	Middle secondary qualifications
			CSE below grade 1/GCSE or O Level below grade C, SCE Standard, Ordinary grades below grade 3 or Junior Certificate below grade C; NVQ or SVQ Level 1/GNVQ Foundation Level or GSVQ Level 1; BTEC, SCOTVEC first or general certificate/SCOTVEC modules; City & Guilds part 1/RSA Stage I,II,III/Junior certificate	Other school qualifications

Our BMI variable was derived from height and weight measurements made by the interviewer in the MCS6 ‘activity monitor, time use and physical measurement’ survey. Z-scores calculated from these measurements were categorized using age and sex specific cut-offs from the Childhood Obesity Working Group of the International Obesity Taskforce (Cole Eur J Clin Nutr. 1990)

The variables describing the 2004 Rural and Urban Area Classification of the participant’s area of residence and proportion of commuters that cycle to work (as per the 2011 Census) in the local area were determined on the basis of the Lower Super Output Area of the participant’s home postcode in MCS sweep 6. The 2004 Rural and Urban Area Classification identifies Lower Super Output Area as belonging to one of the following categories:

1. Urban major conurbation
2. Urban minor conurbation
3. Urban city and town
4. Rural town and fringe
5. Rural village and dispersed

For our analyses these were collapsed into a two-category variable with ‘urban’ (above categories 1-3) and ‘rural’ (above categories 4 and 5) values.

The 2011 Census described the proportion of adults within each Lower Super Output Area who used cycling as their ‘usual main mode of travel to work’ within the following categories:

1. <1 %
2. 1-1.9%
3. 2-2.9%
4. 3-3.9%
5. 4-4.9%
6. 5-5.9%
7. 6-9.9%
8. 10-14.9%
9. >=15%

From these we developed a four-category variable describing the proportion of commuters that cycled to work as: <2%, 2-3.9%, 4-5.9% or ≥6%.
